# Extracts from Remarks on the Standard Operations for Cataract, and Particularly the Methods Proposed by Mooren and Jacobson

**Published:** 1864-09

**Authors:** Julius Homberger

**Affiliations:** Editor Am. Jour. Ophthalmology


### EXTRACTS FROM REMARKS ON THE STANDARD OPERATIONS FOR CATARACT, AND PARTICULARLY THE METHODS PROPOSED BY MOOREN AND JACOBSON.

By Julius Homberger, M. D., Editor Am. Jour. Ophthalmology.

It is one of the most fruitful results of ophthalmological investigation during the last few years, that by combination of iridectomy with the extraction of cataract, the danger of this operation has been so remarkably diminished, that the latest statistics would seem fantastical to any one unacquainted with the rapid strides of our specialty.

The methods of discission and "linear extraction" had, by Von Graefe's genius, become, since about ten years, a valuable resort for cases of soft and half-soft cataract; and both these methods, when applied to those cases only in which they were indicated, and their indications were clearly defined by Von Graefe, may be considered as almost exempt from danger. The customary operations in cases of hard cataract, however, were, up to a still later time—1858—the same as in the beginning of this century. Extraction with a flap downwards or upwards] or sidewards, and the different methods of reclination, depression, etc., were left to the choice of the operator. The former was followed, in a large per centage of cases, by suppuration of the flap and consecutive panophthalmitis; the latter gave, in a still larger number of cases, ill results at a somewhat later period, produced by the action of the depressed crystalline body on the choroid. The operation by extraction being infinitely more difficult as to its execution, and if unsuccessful followed by immediate destruction of the eye, while the other operations proved only fatal after sight had been for a longer or a shorter period restored, but rarely ended with atrophy of the bulb—it was a necessary consequence, that the cowardice of operators made them shrink from extraction, and exert their skill in modifying reclination and inventing methods, every one of which labored under the same difficulty as the original one—viz., the supposed solubility by absorption of the sclerotized hard substance of the cataractous lens.

A number of the better surgeons, observing the frequent occurrence of total and incurable cecity following reclination and similar procedures, and the durability of eyes which had escaped the danger of suppuration after extraction, adopted the latter method, as comparatively safe; but their statistics in extraction were not brilliant enough to compete with the boasted results of the "reclinators," whose patients almost universally went away "cured," and whose statistical tables remained the same when these patients sought relief, after having become blind, not from the same hands who had blinded them. Thus reclinators and extractors finally believed in a theory of absorption and incapsulation, and it was impossible to judge of the merits of the question by the clinical or statistical data. The result of the accumulated experience of both was a kind of compromise; it was asserted that extraction was a safe method if the patients were vigorous and well nourished, if the eyes were not deep-set, etc., etc.; but that in old and decrepit individuals the tendency to suppuration of the cornea was too considerable, or the execution of extraction in patients with deep-set eyes too difficult, so that reclination had in such cases to be considered as preferable.

This compromise is the only explanation of the fact that we find in a statistical table, published in a previous number of this Journal,* stating the results of four of the most successful operators, 469 cases operated on by reclination and kindred methods, to 3,465 operated on by extraction. The per centage of ill successes in those 469 cases was invariably larger than in those where extraction had been performed; but as the selection of reclination was based on circumstances which seemed to render extraction very uncrtain, these bad results of course did not by themselves speak against the value of the operation.

Nevertheless, the ophthalmological community became more and more doubtful about the advisability of reclination and similar operations, and the minds of operators were constantly directed towards the discovery of an operation which would permit the removal of the diseased lens out of the eye, without on the other hand, requiring the formation of so large a flap in the cornea, as extraction.

To these endeavors we owe the operation which is known by the name of linear extraction and Waldau's operation for "spooning out." The former was claimed by its inventor to be only applicable to demi-soft cataracts with a small nucleus, and the latter did not realize, particularly in the common form of senile cataract, the brilliant results predicted by its originator. The experience of oculists universally has shown that Waldau's operation cannot be considered a more safe method than extraction, if the whole substance of the lens is hardened, or in the common forms of hard cataract, though the spoon invented by Waldau for his operation is one of the most valuable instruments in the truss of the ophthalmic surgeon, particularly when he is called upon to extract cataracts.

*No. 5, 1863, p. 241,

For soft cataracts, the linear extraction, as proposed by Graefe, and Waldau's operation, are most valuable methods, and in my belief, the cases are very rare where they are not preferable to the slow method of discission, except in congenital soft cataracts in very young children. In adults, discission ought to be performed but in exceptional cases.

I may also allude here to the successes obtained by iridodesis in congenital layer cataract, dislocation of the lens, stationary cataracts, and partial obscurations of the crystalline body; and without entering into the discussion of this brilliant achievement of ophthalmic surgery, the remark seems to me justifiable, that any operation for removal of the lens is contraindicated if iridodesis is applicable.

If we exclude the soft and demi-soft cataracts requiring Graefe's and Waldau's methods, and in exceptional cases discission, and those forms of anomalies of the lens which indicate iridodesis. and consider the achievements of the last few years in the operation of hard, senile cataracts, which form the principal subject of this article, we have, as already stated, reasons to be proud of the glorious progress of ophthalmic surgery.

Two writers on the subject are entitled to the praise of having diminished in a considerable degree the dangers inherent to extractions. The performance of iridectomy for the purpose of avoiding iritis, and for facilitating the passage of the lens, was the leading idea of Mooren, who, after he had found the difficulty of performing the operation simultaneously with extraction, instituted iridectomy as a preparatory procedure. Jacobson's principal merit is the application of chloroform narcosis in extraction, and perhaps, also, the advice to form the flap in the border of cornea and sclerotic.

The latter proposition is valuable if the diameter of the cornea is small, but it appears to me, according to the experience I have had with Mooren's method, that it is not essential to success; for if, on the one hand, it diminishes the danger of corneal inflammation, it is not so likely to be followed by rapid union as a cut in the substance of the cornea itself. The main feature of Mooren's and Jacobson's operations are, therefore, the combination of iridectomy with extraction, in which both authors concur, and the methodic application of chloroform.

It is true that iridectomy has been performed here and there, and even been recommended by Von Graefe and others in exceptional cases, previous to the appearance of Mooren's valuable monograph; but it cannot be denied that the statistics given by this author, as well as his remarks on the indications of the modified manner of extraction, have effected the more universal adoption of a method which has proved a most fruitful addition to our means of cure in cataract.

Of more, or at the least of equal, importance as the combination of iridectomy, is the performance of the operation while the patient is in a state of perfect muscular rest. I believe that even if Dr. Jacobson would have extracted without iridectomy, in his 100 cases the results would have been also very good, though the losses would surely not have attained the comparatively small number of 2 per cent.

The advice of Jacobson to replace the iris if it prolapses after the completion of the flap, does not seem to me as rational as the performance of iridectomy as the second act of the operation, if such an accident happens. The hemorrhage which follows in such cases the excision of the iris is very slight, and it is hardly necessary to see the pupil clearly in order to open the capsule with the cystotome, or to remove the lens in the customary manner. Besides, as in both these steps of the operation the flap is opened, the aqueous humor, with the effused blood. escape, and allow a fair view of the pupillary space, etc. I have in two cases, in both of which the lower segment of the iris had been lacerated during the completion of the flap thus performed iridectomy before opening the capsule, and the results have been equally good as in the normal operations. The same course is advisable, as Jacobson himself remarks, if the lens cannot be easily delivered by the pressure of the finger, which must be particularly frequent if there exist synechies, as I found it in one case, or if the vitreous is abnormally fluid, as in cataracts in persons with myopia and scleriectasia posterior. In the latter condition it is evident that this rule ought always to be adhered to, because the delivery of the lens can be caused by a smaller amount of pressure as if it has to be forced through a central pupil. The effusions of blood into the anterior chamber, and hemorrhages from the vessels of the conjunctiva, are without any injurious influence on the success of operations.

The careful application of a compressive bandage, with all the cautions which may be [exerted in order to envelop the eye with a homogeneous pressure, is perhaps as essential to success, and more so, than a normal and skillful performance of the operation itself. I do not hold that the careful nursing which Jacobson considers so essential is superfluous; but I had frequently to operate under circumstances which would not permit so careful a treatment, and as in other cases the directions I had given in this respect were disregarded, I believe that, *ceteris paribus*, a patient operated on with chloroform will not require more careful attendance than if operated on in another method, provided the bandage is well applied. I do not use the buckled bandages, which are so much used now, and recommended by Jacobson, but a flannel bandage, 5 to 6 yards long, which I apply, after the lint is well put on both eyes, in the form of the binoculus. I also instructed my patients to lay their flat hands themselves on the bandage while vomiting, instead of intrusting this to the nurse. Intelligent patients will easily understand the reason for thus supporting their eye, and perform this in an adequate manner. I have never yet had a reason for applying cold water compresses, nor any other antiphlogistic, after operations.

The after-treatment with atropine is of great value, without doubt. I apply it in substance once daily, and have in each case succeeded in dilating the pupil from the third day, as far as it is possible after a piece of the iris has been removed. I believe that the application of atropine in substance is superior to instillations, as the doses given suffices for a period of 24 hours, and as it is possible to continue the use of the bandage.

